# Readability analysis of breast cancer resources shared on X—implications for patient education and the potential of AI

**DOI:** 10.1007/s10549-025-07799-z

**Published:** 2025-08-06

**Authors:** Melanie J. Wang, Aref Rastegar, Theodore A. Kung

**Affiliations:** Department of Surgery, Section of Plastic Surgery, University of Michigan, Ann Arbor, MI USA

**Keywords:** Breast cancer, Health literacy, Online health information, Patient education, Readability

## Abstract

**Purpose:**

Breast cancer remains a global public health burden. This study aimed to evaluate the readability of breast cancer articles shared on X (formerly Twitter) during Breast Cancer Awareness Month (October 2024), and it explores the possibility of using artificial intelligence (AI) to improve readability.

**Methods:**

We identified the top articles (*n* = 377) from posts containing #breastcancer on X during October 2024. Each article was analyzed using 9 established readability tests: Automated Readability Index (ARI), Coleman-Liau, Flesch-Kincaid, Flesch Reading Ease, FORCAST Readability Formula, Fry Graph, Gunning Fog Index, Raygor Readability Estimate, and Simple Measure of Gobbledygook (SMOG) Readability Formula. The study categorized sharing entities into five groups: academic medical centers, healthcare providers, government institutions, scientific journals, and all others. This comprehensive approach aimed to evaluate the readability of breast cancer articles across various sources during a critical awareness period of peak public engagement. A pilot study was simultaneously conducted using AI to improve readability. Statistical analysis was performed using SPSS.

**Results:**

A total of 377 articles shared by the following entities were analyzed: academic medical centers (35, 9.3%), healthcare providers (57, 15.2%), government institutions (21, 5.6%), scientific journals (63, 16.8%), and all others (199, 53.1%). Government institutions shared articles with the lowest average readability grade level (12.71 ± 0.79). Scientific journals (16.57 ± 0.09), healthcare providers (15.49 ± 0.32), all others (14.89 ± 0.17), and academic medical centers (13.56 ± 0.39) had higher average readability grade levels. Article types were also split into different categories: patient education (222, 58.9%), open-access journal (119, 31.5%), and full journal (37, 9.6%). Patient education articles (15.19 ± 0.17) had the lowest average readability grade level. Open-access and full journals had an average readability grade level of 16.65 ± 0.06 and 16.53 ± 0.10, respectively. The mean values for Flesch Reading Ease Score are patient education 38.14 ± 1.2, open-access journals 16.14 ± 0.89, full journals 17.69 ± 2.14. Of note, lower readability grade levels indicate easier-to-read text, while higher Flesch Reading Ease scores indicate more ease of reading. In a demonstration using AI to improve readability grade level of 5 sample articles, AI successfully lowered the average readability grade level from 12.58 ± 0.83 to 6.56 ± 0.28 (*p* < 0.001).

**Conclusions:**

Our findings highlight a critical gap between the recommended and actual readability levels of breast cancer information shared on a popular social media platform. While some institutions are producing more accessible content, there is a pressing need for standardization and improvement across all sources. To address this issue, sources may consider leveraging AI technology as a potential tool for creating patient resources with appropriate readability levels.

## Introduction

Breast cancer remains a significant global health concern, warranting focused attention in health communication research. According to the World Health Organization, breast cancer is the most common cancer among women worldwide, with an estimated 2.3 million new cases diagnosed in 2022 [1]. The impact of breast cancer is particularly stark when considering mortality rates: approximately 670,000 women died from breast cancer globally in 2022 [1]. In the United States alone, it is projected that 316,950 new cases of invasive breast cancer will be diagnosed in women in 2025 [2] Social media platforms have emerged as powerful tools in this context, with studies [3] showing their potential to increase awareness about modifiable breast cancer risk factors and promote cancer screening participation. However, the effectiveness of these platforms in disseminating accurate, comprehensible health information varies significantly.

X (formerly Twitter) in particular has emerged as a prominent social media platform [4]. Social media sites such as X can be effective in delivering health information and issuing medical alerts because they are easy to use and can provide real-time updates. For instance, in the 2009 H1N1 outbreak, local health departments and federal health agencies turned to social media to share reports of vaccination availability, flu clinic hours, and fatality numbers [5, 6]. X is used differently by various health organizations (i.e., CDC, National Institutes of Health, major medical centers). The fact that 12% of people use X or another site to get updates about health concerns supports the value of X as a helpful outlet for health communication [7]. Moreover, studies have shown that individuals frequently seek online health information before consulting a healthcare provider. In one study analyzing video outreach on a social media platform, the median number of views per video was over 97,000, with some videos viewed more than 1.9 million times, demonstrating the vast reach and influence of online health content [8]. These findings emphasize the pivotal role that online health information plays in shaping patient behavior and access to care. However, it is critical that this information shared with the public is at a level of comprehension that is appropriate for the target audience. Specifically in plastic surgery, analyses of patient resources on topics like breast reconstruction [9, 10], lymphedema [11], and breast cancer [12] have revealed that the information is often too complex for patients to easily understand. [13, 14]

Health literacy is the ability to access, understand, and use health information to make well informed health decisions [15]. A 2003 National Assessment of Adult Literacy [16] showed that the average reading level of most Americans was at the level of 7-8th graders, equal to a 12- to 13-year-old child with 7 to 8 years of schooling. Correspondingly, the American Medical Association (AMA) and the National Institutes of Health (NIH) guidelines states that patient education materials should not exceed a sixth grade reading level [17]. Despite these recommendations, studies have found that the web-based and text-based health information tools designed for the public are often too complex and difficult to use.

In this study, we looked at how various entities shared articles relating to breast cancer on X during Breast Cancer Awareness Month (October 2024). Our goal was to analyze the readability of resources shared on this social media platform and explore the possibility of using artificial intelligence (AI) to improve readability. This research provides valuable insight into the evolving role of social networking sites as health communication tools and how health organizations can use these platforms effectively.

## Methods

### Data collection

For data collection, X (formerly Twitter) was used to extract top posts using an algorithm designed by X. The data collection was completed independently by two study authors (MJW, AR) to ensure reproducibility of the data and reported values for the analysis. An Internet search was conducted for all top tweets containing #BreastCancer on X in October of 2024. The cross-sectional study specifically focused on October 2024, as it coincides with Breast Cancer Awareness Month, a globally recognized observance aimed at increasing attention and support for breast cancer awareness, early detection, and treatment [18]. The decision to concentrate on this month was strategic, with the aim of evaluating whether the focus on breast cancer during this dedicated period resulted in the dissemination of information that was genuinely readable by the general public. This approach allowed for an assessment of the readability of breast cancer information during a time when public engagement with the topic is typically at its peak.

The search was conducted after turning off filters and disabling location, cookies, and user account information to minimize bias, as previously performed by other Web-based search studies [19, 20]. Articles were downloaded into plain text in separate Microsoft Word 2011 documents (Microsoft Corp., Redmond, Wash.). These texts were then edited to exclude any hyperlinks, advertisements, images, videos, figures, links, or extraneous information. We categorized articles into three groups: (1) patient education materials (PEM), (2) open-access journal articles, and (3) full access (subscription-based) journal articles. This stratification was designed to assess potential readability differences across materials intended for direct patient consumption (PEM), materials freely available to the public (open-access), and materials that require institutional or individual subscription (full text). Although open-access articles are also full text, the distinction was made to compare the readability of materials designed for broad, barrier-free dissemination (open-access) with those potentially targeted toward an academic or professional audience (subscription-based full access). This categorization allowed us to evaluate if accessibility (open vs. subscription) and intended audience (patient-directed vs. professional) influenced readability metrics. Five different account types were extracted based on the account that shared the X post: (1) academic medical centers; (2) healthcare providers (medical professionals, clinics, and community organizations); (3) government institutions; (4) scientific journals; (5) ‘all other’ (public, misc.).

### Readability Analysis

Readability analysis was performed using Readability Studio Professional Edition (Oleander Software Ltd; Vandalia, OH), which is a widely used software that assesses the readability of health information [21–23]. Readability grade level was evaluated using 9 established tests: Automated Readability Index (ARI), Coleman-Liau, Flesch-Kincaid, Flesch Reading Ease, FORCAST Readability Formula, Fry Graph, Gunning Fog Index, Raygor Readability Estimate, and Simple Measure of Gobbledygook (SMOG) Readability Formula **(**Table 1**)**.

**Table 1:** List of readability formulas provided by Readability Studio Professional Edition

Readability tool	Formula	Description
Automated readability index (ARI)	4.71 (# of letters and numbers/# of spaces) + 0.5 (# of spaces/# of sentences) – 21.43	ARI was originally developed to ensure that the technical reports and manuals for U.S. Air Force materials are easy to read. It uses the number of characters per word and the number of words per sentence to calculate reading grade level
Flesch-kincaid	(0.39 x # of “easy” words within sample) + (11.8 × average # of words per sentences) – 15.59	Flesch-Kincaid test is used to ensure technical documents and manuals are easy to read and comprehend for Navy officers. It uses the number of syllables per word and the number of words per sentence to estimate the appropriate reading grade level
FORCAST (Ford, Caylor, Stitch) readability formula	20 – (# of single syllable words in a 150 word sample ÷ 10)	FORCAST readability test was developed for U.S. Army technical manuals and forms. It considers the number of monosyllabic words in the text to determine the reading grade level
Gunning fog index	0.4 x ((average # of words per sentences ÷ average number of sentences) + 100 (average number of words with > 3 syllables ÷ average # of words per sentences))	The Gunning Fog readability formula calculates the grade level of a document based on its number of sentences and complex words (i.e., three or more syllables)
Fry graph	Select three 100-word passages from beginning, middle, and end of a text. Calculate the average # of sentences and syllables in each passage. Plot on Fry Graph to determine reading level	Fry readability test calculates a document’s grade level from its average number of sentences and syllables per hundred words
Raygor readability estimate	100 words passage selected from text. # of sentences and # of words with > 6 letters calculated. Assessed on graph and readability measured	Raygor’s Readability Estimate calculates a document’s grade level from its average number of sentences and long (6 + character) words per hundred words
Simple measure of gobbledygook (SMOG)	1.043 x √ (H x (30/average number of sentences)) ÷ 3.1291	SMOG Readability Formula calculates the grade level of a document based on complex (i.e., 3 or more syllable) word density
Coleman-liau	(0.0588 (average # of letters per 100 words) – 0.296 (average # of sentences per 100 words)) – 15.8	Coleman and Liau highlighted the challenges associated with using computers for syllable counting in readability assessments. In response, they developed a new formula that utilizes character counts instead. The Coleman-Liau formula determines the document’s grade level and estimated cloze score by considering the length of sentences and the character count
Flesch reading ease	206.835 – (1.015 x # of “easy” words within sample)	Uses a unique 0–100-point scale where higher scores indicate easier readability

Each readability test uses a unique formula to estimate the minimum U.S. grade education required for a person to be able to read and understand the text [24]. Most grade-level tests can score up to grade 19. All readability test results were averaged together to depict the likely necessary reading grade level to understand the posted literature, except for the Flesch Reading Ease score. This approach was taken because the Flesch Reading Ease score uses a unique 100-point scale where higher scores indicate easier readability. In contrast, with the other readability scores, lower readability grade levels indicate easier-to-read text. We modeled our methods of averaging readability scores based on established approaches in previous research [25, 26]. The readability grade levels indicate the educational stage required to understand a text, ranging from kindergarten (grade 0) to doctoral levels (grade 19 +). The numbers correlate with the grade level. Texts with readability grade level 0 to 12 are designed for students from early schooling to high school. Texts beyond readability grade level 12 are intended for higher education, including college level (13–16), graduate level (17–18), and post-graduate/doctorates (19 +), demanding specialized knowledge and advanced reading abilities.

### Pilot study

A pilot study was simultaneously conducted to assess the ability of AI to improve the readability of patient education materials. Five breast reconstruction surgery and breast cancer patient education articles were randomly selected, all of which exceeded the 6th-grade readability level recommended by the American Medical Association (AMA) and the National Institutes of Health (NIH). The original text of each article was input into ChatGPT (OpenAI, San Francisco, CA, USA) with instructions to lower the readability grade level while preserving key information. Subsequently, the AI-generated versions were evaluated using the same set of 9 established readability tests employed in the main study to assess the effectiveness of the readability improvement.

### Statistical analysis

The mean overall reading grade level was calculated for both categories and compared using statistical analysis in Statistical Package for the Social Sciences (SPSS) (IBM Corp, Armonk, NY, USA). The data were treated as nonparametric due to their distribution and analyzed using the Kruskal–Wallis test to compare medians across multiple groups. For post hoc analysis, Dunn’s test with a Bonferroni correction was utilized to adjust for multiple comparisons, with the significance threshold set at *p* < 0.005. Results are presented as mean + SEM (Standard Error of the Mean).

## Results

### Types of articles shared

**Fig. 1:**
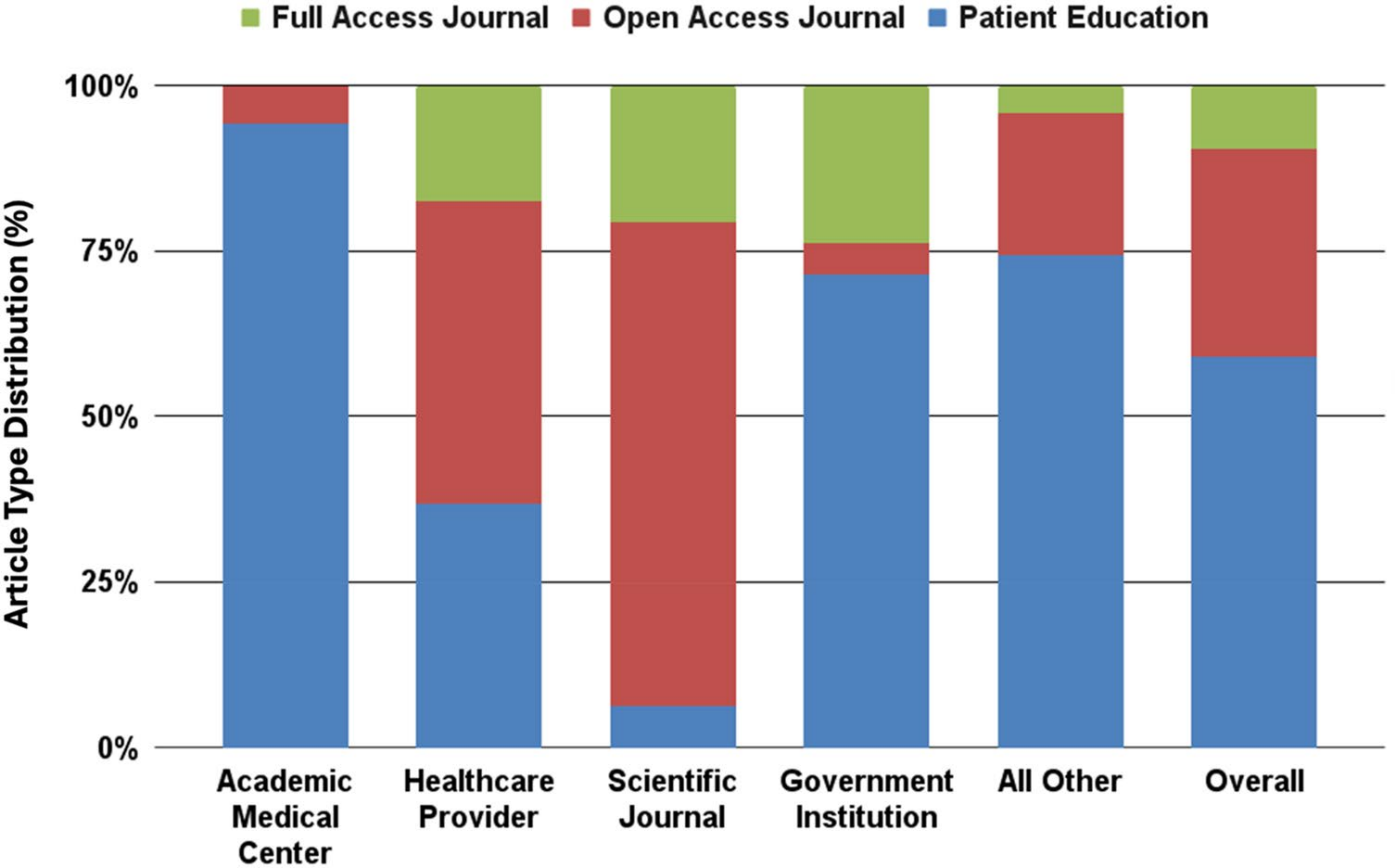
Distribution of articles shared by different account types

Of the total articles (n = 377) shared across X, academic medical centers contributed 35 (9.3%), healthcare providers 57 (15.2%), government institutions 21 (5.6%), scientific journals 63 (16.8%), and ‘all other’ sources accounted for 199 (53.1%) (Fig. 1). It should be noted that these articles were not authored by the sharing entities but were merely shared (tweeted) by them. Academic medical centers shared 2 (5.7%) open-access journals and 33 (94.3%) patient education articles. Healthcare providers shared 26 (45.6%) open-access, 10 (17.5%) full journal, and 21 (36.8%) patient education articles. Government institutions shared 1 (4.8%) open-access, 5 (23.8%) full journal, and 15 (71.4%) patient education articles. Scientific journals shared 46 (73.0%) open-access, 13 (23.6%) full journal, and 4 (6.3%) patient education articles. ‘All other’ sources shared 43 (21.6%) open-access, 8 (4.0%) full journal, and 148 (74.4%) patient education articles.

### Readability grade level by account category

**Fig. 2:**
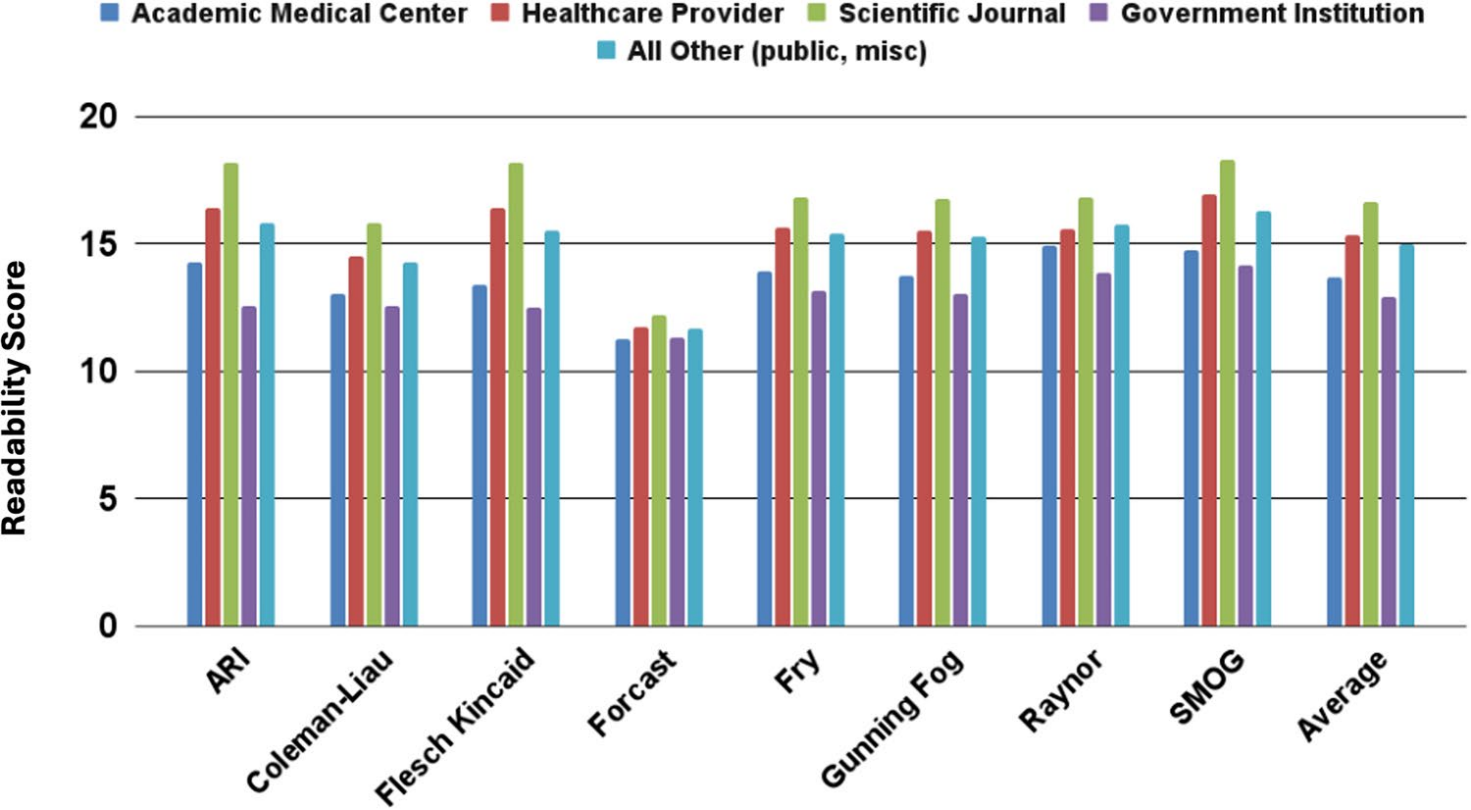
Readability breakdown by test for articles posted by different account types. ARI, Automated Readability Index; SMOG, Simple Measure of Gobbledygook Readability Formula

Our analysis provided a detailed assessment of readability scores across various tests and account categories, as summarized in Fig. 2. We separated the data into two metrics: average readability grade level and Flesch Reading Ease score.

### Average readability level

**Fig. 3:**
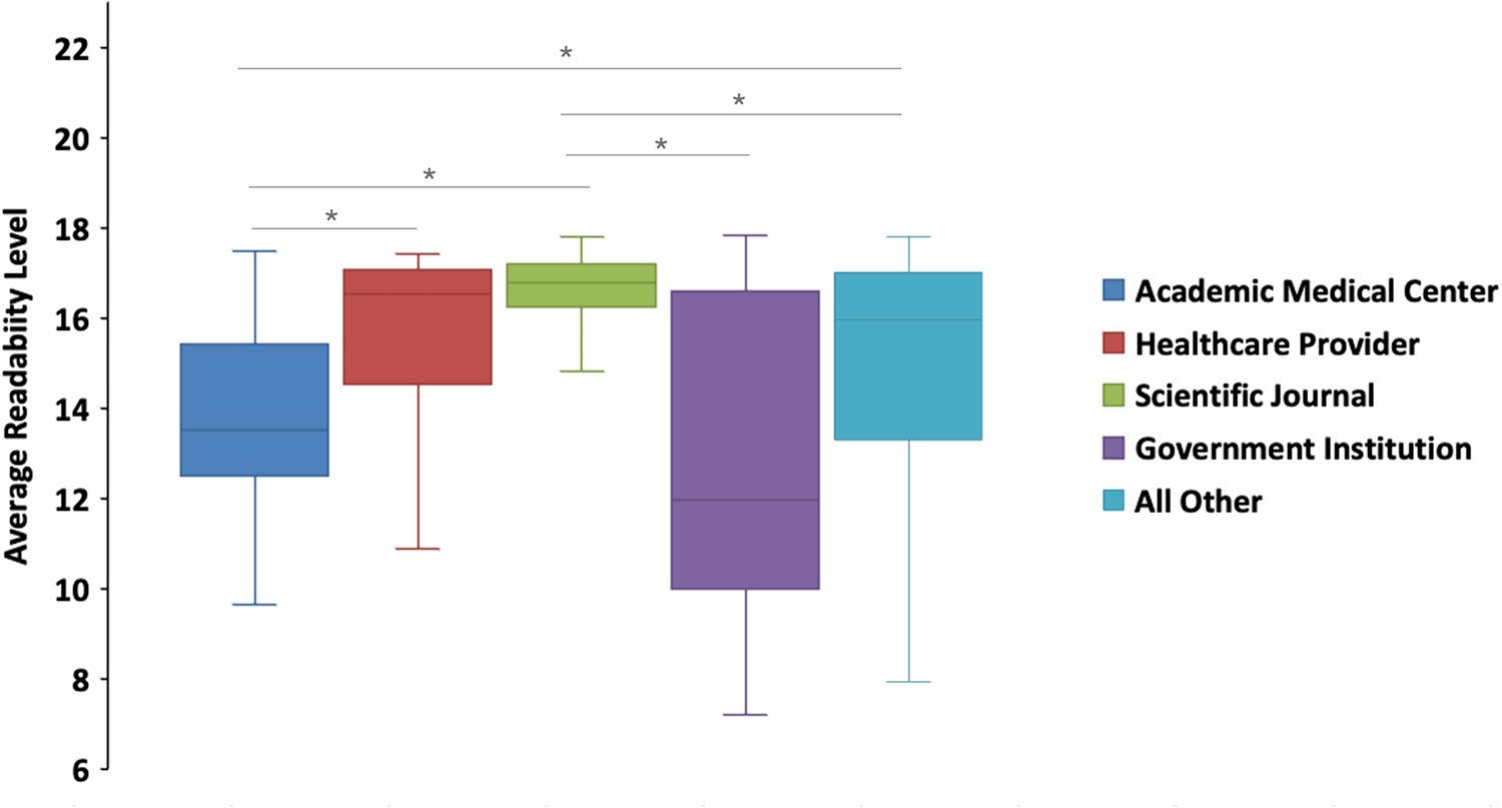
Average readability grade level across different account types

The average readability level for each category (Fig. 3) varied considerably. Scientific journals had the highest average readability score at 16.57 ± 0.09, indicating content most appropriate for a graduate-level audience. In contrast, government institutions had the lowest average readability score at 12.71 ± 0.79, suggesting texts that are comparatively easier to read. The remaining categories fell between these two ends, with healthcare providers averaging 15.49 ± 0.32, academic medical centers at 13.56 ± 0.39, and ‘all other’ accounts at 14.89 ± 0.17.

### Flesch reading ease

**Fig. 4:**
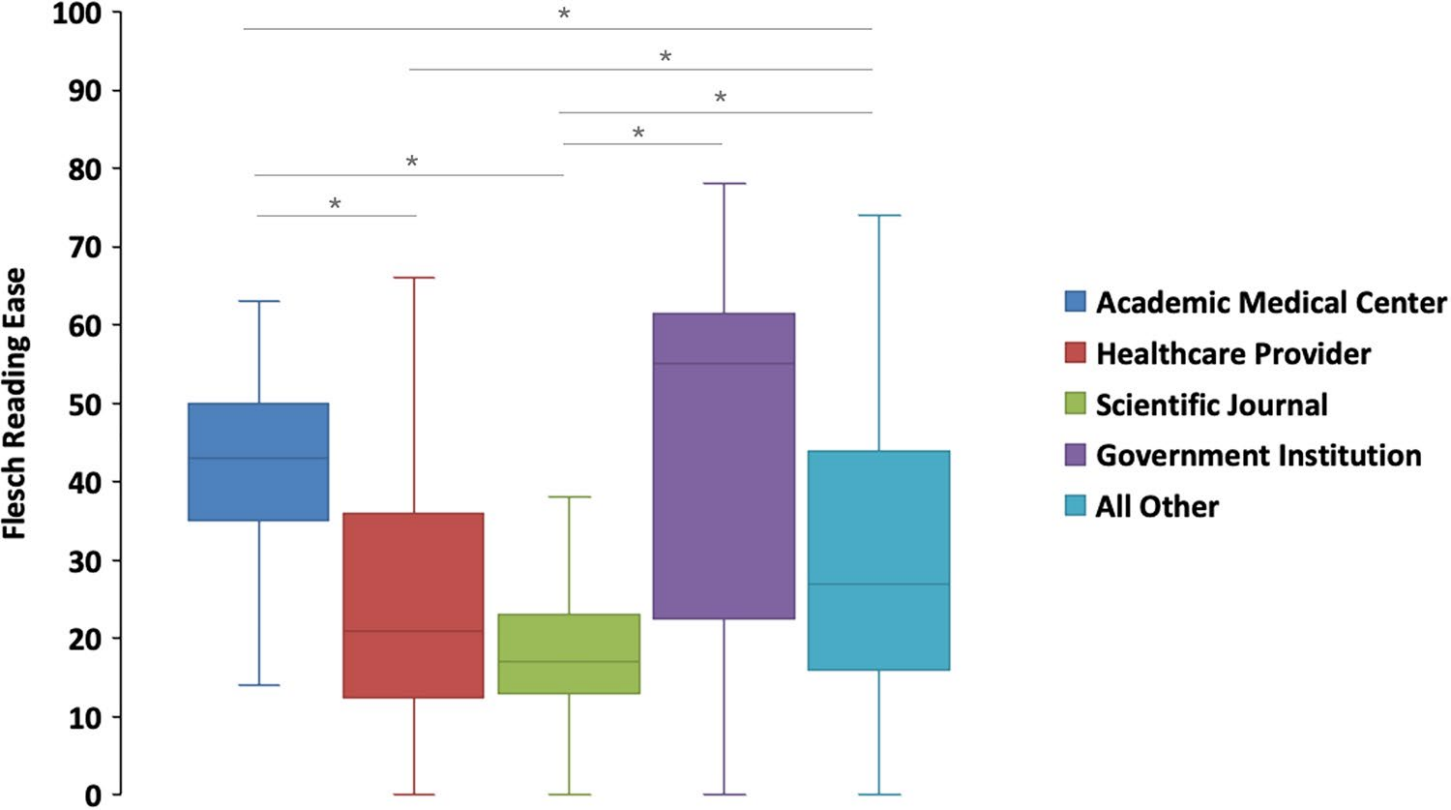
Flesch reading ease score across different account types

The mean values for Flesch Reading Ease based on the account category (Fig. 4) are as follows: academic medical centers (40.77 ± 2.66), scientific journals (17.49 ± 1.28), government institutions (42.76 ± 5.55), healthcare providers (26.29 ± 2.60), and ‘all other’ (30.34 ± 1.25). Flesch Reading Ease scores were found to be congruent with the average readability levels, exhibiting analogous patterns across the different groups.

### Readability grade level by article type

**Fig. 5:**
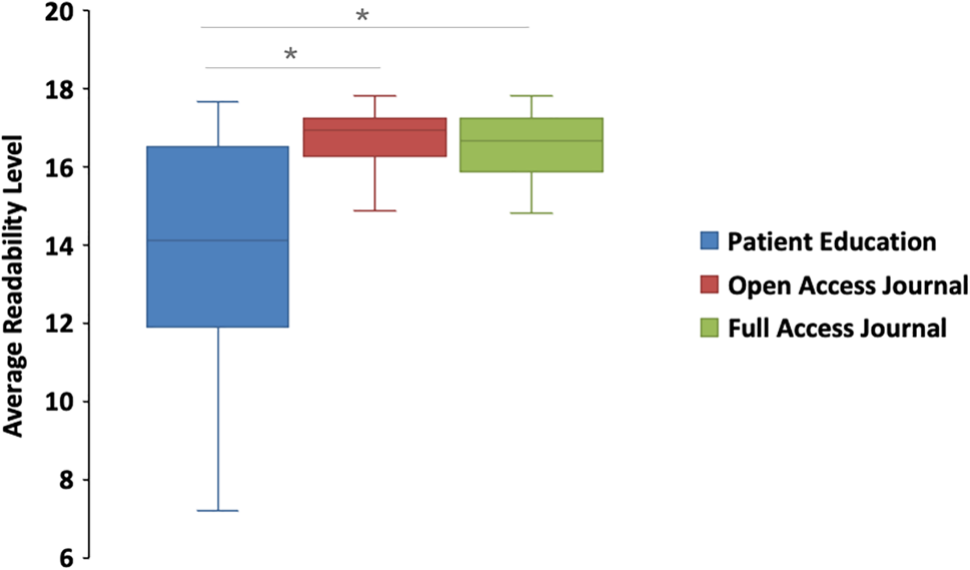
Average readability grade level by article types

**Fig. 6:**
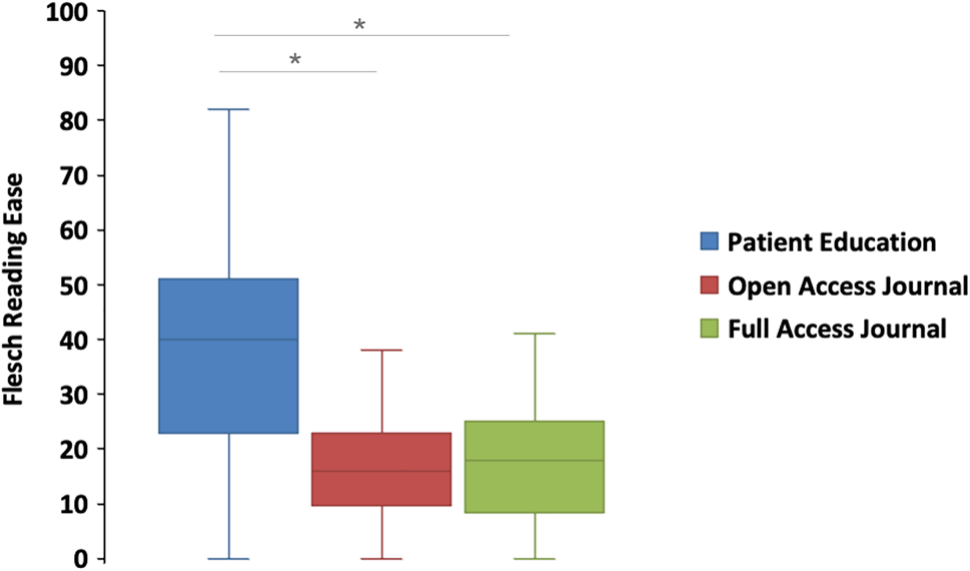
Flesch reading ease by article types

A total of 377 articles were extracted from X in October 2024, including 119 (31.5%) open-access journal, 37 (9.6%) full journal, 222 (58.9%) patient education journal. The mean values for average readability grade level (Fig. 5) are as follows: patient education (15.19 ± 0.17), open-access journals (16.65 ± 0.06), full journals (16.53 ± 0.10). Similarly, the mean values for Flesch Reading Ease score (Fig. 6) are patient education 38.14 ± 1.2, open-access journals 16.14 ± 0.89, full journals 17.69 ± 2.14.

### Artificial Intelligence Pilot Study

The original readability scores (Table 2) for the articles were Patient Education Article 1 (14.6), Patient Education Article 2 (12.3), Patient Education Article 3 (10.6), Patient Education Article 4 (11), Patient Education Article 5 (14.4). After processing with ChatGPT, these scores improved to 7, 6.2, 5.7, 6.7, and 7.3, respectively. Overall, AI successfully lowered the average readability grade level from 12.58 ± 0.83 to 6.56 ± 0.28 across the five articles (*p* < 0.001).

**Table 2:** Patient education articles before and after AI

Article	Original grade level	ChatGPT grade level
Patient Education Article 1	14.6	7
Patient Education Article 2	12.3	6.2
Patient Education Article 3	10.6	5.7
Patient Education Article 4	11	6.7
Patient Education Article 5	14.4	7.3
**Mean grade level**	12.58 ± 0.83	6.56 ± 0.28
**p-value**	P < 0.001	

## Discussion

The most significant finding of our study is that the average readability grade level of sampled breast cancer-related materials is substantially higher than what is recommended for public consumption across all categories. Our results reveal that patient education materials, while more readable than open-access and full journal articles, still have an average grade level of 13.56 ± 0.39, far exceeding the 6th to 8th grade level guidelines set by the AMA and NIH. Government organizations on average produce more readable content compared to academic medical centers and healthcare providers, but a mean readability grade level of 12.71 ± 0.79 for government organizations is still too high. Other studies have found similar results and point to the ongoing challenge of making health information truly accessible for breast cancer patients. Vargas et al. (2014), for example, examined 114 online resources on breast reconstruction from academic and non-academic sources and found that average readability levels were between the 11th and 13th grade. That’s still way above the 6th-grade level recommended by the NIH and AMA. They observed that materials from academic medical centers were more complex than those from other health-related websites, which is in line with our findings [27]. Cortez et al. (2015) evaluated readability of online breast cancer risk assessment tools and reported a mean SMOG readability grade level of 12.1, despite the fact that these resources are specifically intended to support patient decision-making [28]. Overall, these studies suggest that despite the emphasis on health literacy in recent years, most institutions still use language that is too advanced for the general public. These findings have several important implications. High readability grade levels from all posting entities may significantly impair patients’ ability to understand crucial health information. Breast cancer patients face a complex landscape of receiving medical information, from diagnosis through treatment, reconstruction, and recovery. Their ability to comprehend and apply this information directly impacts their participation in shared decision-making processes with healthcare providers, adherence to treatment plans, and overall health outcomes. [29]

This isn’t just a problem in breast cancer. Poor readability comes up across other areas of medicine, too. For instance, Kiuchi et al. (2025) looked at patient education materials from different medical fields and found that 99% of them went above the 6th-grade readability level, and many were higher than the 10th grade [30]. Özduran (2022) found the same issue in Turkish-language materials on low back pain and stroke rehabilitation and —those also exceeded recommended readability levels [31, 32]. Specifically in plastic surgery, analyses of patient resources on topics such as breast reconstruction, lymphedema, and breast cancer have consistently shown that the readability of available materials is often too complex for the average patient to understand [9–14]. These examples highlight that the problem is not limited to one specialty or language.

Despite public guidelines for writing health education materials, readability levels of web-based health resources, even those written by public health organizations and major academic institutions on breast cancer and breast reconstruction have often remained challenging for patients to understand [27, 33]. The lack of standardization in patient education materials across institutions highlights the need for more stringent guidelines and quality control measures. There appears to be a gap between producers of scientific knowledge and those responsible for public health communication. The complexity of shared content may exacerbate health literacy issues, particularly among vulnerable populations. To address these findings, future efforts may include the development of standardized readability guidelines for patient education materials, collaboration between researchers and health communicators, and the use of AI-based tools to simplify complex medical content while preserving accuracy.

AI presents a promising solution to address the challenge of creating health-related content that meets recommended readability levels [34]. By leveraging AI tools, healthcare organizations could potentially improve the readability of patient education materials, ensuring they are comprehensible to a wider audience. To illustrate this potential, we conducted an experiment using ChatGPT to improve the readability of randomly selected patient education articles. These 5 breast cancer and breast reconstruction patient education articles had readability grade levels that exceeded the 6th-grade level recommended by the AMA and the NIH. The results showed an improvement in readability grade level across all articles. This experiment demonstrates the potential of AI in enhancing the readability of health information. Future implementations could involve integrating AI-assisted readability checks as a standard practice before publishing content on social media or other platforms. By adopting these technologies and establishing standardized processes for educational content generation, the healthcare community can work toward ensuring that critical health information is universally understandable and accessible, potentially leading to better-informed patients and improved health outcomes.

## Limitations

This study is limited by the fact that we focused on data from a single month, which may not capture potential seasonal variations or long-term trends in readability. Future studies could expand on this by examining readability changes over extended periods, potentially uncovering patterns related to awareness months, policy changes, or evolving communication strategies. Additionally, our analysis was limited to English-language content, which may not reflect the readability of materials in other languages or for diverse cultural contexts. Future research could explore cross-linguistic comparisons and cultural adaptations of breast cancer information. While we assessed readability, we did not evaluate the comprehensibility, quality, or reliability of the information provided. However, we note that high readability does not inherently compromise quality or reliability. As demonstrated in similar work evaluating YouTube health content, readability and reliability can coexist when information is curated by reputable sources [35]. Future studies could combine readability analyses with comprehensibility and quality assessments for a more comprehensive evaluation. One important consideration in interpreting our results is the inclusion of scientific journal articles in our readability analysis. It is well established that scientific publications are written for a professional audience and therefore tend to have higher readability grade levels. While our goal was to capture the full spectrum of information shared with the public during Breast Cancer Awareness Month, including journal articles, this inclusion may have contributed to overall higher average readability scores. Some studies addressing similar topics have chosen to exclude scientific journals entirely to avoid this confounding factor [36]. We acknowledge this in our study and advise readers to interpret results accordingly.

## Conclusion

In conclusion, the average readability grade level of breast cancer-related content shared on X during Breast Cancer Awareness Month is significantly higher than recommended across all categories. While some institutions shared patient education resources that were easier to read than others, there remains a substantial need for improvement across the board. Moving forward, healthcare organizations may prioritize the development of standardized processes for generating easily comprehensible health information, potentially leveraging AI-powered tools to ensure content meets recommended readability levels.

## Data Availability

No datasets were generated or analysed during the current study.
